# Live-cell imaging reveals impaired detoxification of lipid-derived electrophiles is a hallmark of ferroptosis†

**DOI:** 10.1039/d2sc00525e

**Published:** 2022-08-01

**Authors:** Antonius T. M. Van Kessel, Ryan Karimi, Gonzalo Cosa

**Affiliations:** Department of Chemistry, McGill University 801 Sherbrooke Street West Montreal Quebec H3A 0B8 Canada gonzalo.cosa@mcgill.ca +1-514-398-3797 +1-514-398-6932

## Abstract

The central mechanism in ferroptosis linking lipid hydroperoxide accumulation with cell death remains poorly understood. Although lipid hydroperoxides are known to break down to reactive lipid-derived electrophiles (LDEs), the ability of cells to detoxify increasing LDE levels during ferroptosis has not been studied. Here, we developed an assay (ElectrophileQ) correlating the cellular retention *vs.* excretion of a fluorogenic lipophilic electrophile (AcroB) that enables live-cell assessment of the glutathione-mediated LDE conjugation and adduct export steps of the LDE detoxification pathway. This method revealed that during ferroptosis, LDE detoxification failure occurs through decreased conjugation or export impairment, amplifying cellular electrophile accumulation. Notably, ferroptosis susceptibility was increased following exacerbation of LDE-adduct export impairment through export channel inhibition. Our results expand understanding of the ferroptosis molecular cell death mechanism to position the LDE detoxification pathway as a ferroptosis-relevant therapeutic target. We envision the ElectrophileQ assay becoming an invaluable tool for studying ferroptosis and cellular health.

## Abstract 2

A link between altered lipid-derived electrophile (LDE) metabolism during ferroptosis and associated cell death was uncovered using a new imaging method developed to monitor cellular LDE detoxification that employs a fluorogenic LDE analogue.

## Graphical abstract

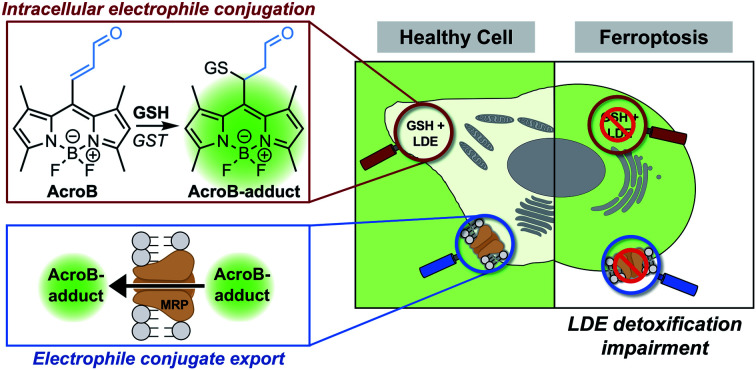

## Introduction

**Fig. 1:**
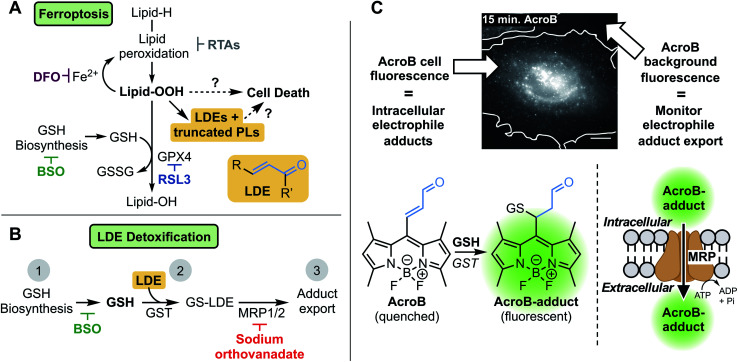
Investigating the role of lipid-derived electrophile (LDE) detoxification in ferroptosis. (A) LDE and truncated phospholipid (PL) accumulation is a promising link between lipid hydroperoxide production and cell death in ferroptosis. Key relevant steps in ferroptosis involve high levels of lipid oxidation following induction through either GPX4 inhibition (RSL3) or GSH depletion (BSO). Iron chelation (DFO) or radical-trapping antioxidants (RTAs) prevent ferroptotic cell death. (B) LDE detoxification occurs through three steps: (1) GSH biosynthesis (inhibited by BSO), (2) GSH conjugation catalyzed by GST, and (3) active export through MRP channels (inhibited by sodium orthovanadate). (C) Widefield fluorescence imaging with AcroB enables monitoring of electrophile detoxification ability through quantification of both cellular fluorescence (fluorogenic AcroB retention, reporting on intracellular electrophile conjugation and level of intracellular electrophile adducts) and background fluorescence (AcroB active export/excretion, reporting on cellular electrophile adduct export). Widefield fluorescence image of a HeLa cell 15 minutes after addition of 100 nM AcroB, white line indicates cell border determined from complementary DIC image. Image acquired at 100× magnification, *λ*_ex_ = 488 nm (0.05 mW), scale bar is 12 μm, LUT range 0–5000.

Ferroptosis is a form of necrotic cell death characterized by high levels of iron-dependent lipid peroxidation and lipid hydroperoxide (LOOH) accumulation.^1^ The identification of both ferroptosis susceptibility in cancer^2–4^ and ferroptotic cell death in degenerative diseases^5–7^ has established this cell death pathway as a promising therapeutic target.^8–10^ Several mechanisms of both ferroptosis induction and inhibition have been identified (Fig. 1A).^9^ Ferroptosis induction strategies include covalent (RSL3) or indirect inhibition (glutathione, GSH, depletion) of GSH-dependent glutathione peroxidase 4 (GPX4), the enzyme responsible for detoxification of LOOH to lipid alcohols (LOH).^11,12^ Prevention of ferroptosis often relies on strategies to decrease lipid peroxidation, namely through use of iron chelators or radical-trapping antioxidants (RTAs).^8^

Despite thorough mapping of ferroptosis regulatory pathways upstream of LOOH accumulation, the ferroptosis cell death mechanism is poorly understood.^8–10,13–18^ Downstream of lipid peroxidation, plasma membrane LOOH accumulation^19^ eventually results in the formation of small membrane pores that facilitate cell swelling, calcium influx and wave-like ferroptosis propagation before complete cell rupture.^17,18^ The breakdown of LOOHs to diffusible lipid-derived electrophiles (LDEs) and truncated phospholipids (PLs) represents a promising link between LOOH accumulation, membrane permeabilization and cell death in ferroptosis.^13–16,20–22^ LDE increase following LOOH accumulation has been characterized during ferroptosis^23–26^ while the upregulation of specific aldo-ketoreductases, the class of enzymes responsible for reduction of LDEs to their less reactive alcohols, has been observed and is associated with partial ferroptosis resistance.^27,28^

LDEs are highly reactive species capable of protein and DNA alkylation as well as cellular signalling pathway activation, while truncated PLs are known to impair lipid membrane integrity and are probable candidates to mediate membrane permeabilization in ferroptosis.^29–32^ In healthy cells, LDE levels are maintained through detoxification *via* GSH conjugation catalyzed by the glutathione *S*-transferase (GST) class of enzymes followed by active export of the GS-LDE adducts through multidrug resistance protein (MRP) channels (Fig. 1B).^33,34^ This mechanism works complementary to the aforementioned reduction of LDEs by aldo-ketoreductases. The elevated LDE production during ferroptosis along with the toxic nature of these reactive metabolites necessitates investigation of the potential role LDE detoxification plays in ferroptotic cell death.^23–26^

To investigate LDE metabolism in ferroptosis, we developed the Electrophile detoxification Quotient (ElectrophileQ) assay. This assay employs a previously reported fluorogenic (turn-on) electrophile probe, AcroB, to enable real-time monitoring of both the critical conjugation and export steps of LDE detoxification in live cells (Fig. 1C).^35^ Prior to this study, the final stages of ferroptosis were largely characterized by LOOH buildup and loss of cell membrane integrity.^1,9,17,18^ Application of the ElectrophileQ assay, in combination with relative quantification of cellular electrophiles, establishes LDE detoxification impairment associated with electrophile accumulation as a hallmark of ferroptosis downstream of LOOH accumulation. Additionally, through linking impaired LDE detoxification with increased cell death susceptibility following ferroptosis induction, we position LDE detoxification failure as both a potential critical stage in ferroptosis pathology and a possible therapeutic target.

## Results

### Real-time monitoring of LDE detoxification

Our investigation of the LDE detoxification pathway in ferroptosis began with the development of a method to observe both cellular LDE conjugation and adduct export in live cells using the fluorogenic electrophile AcroB (Fig. 1C). AcroB was designed as an LDE mimic, containing the reactive α,β-unsaturated aldehyde warhead characteristic of many LDEs appended to a reporting, lipophilic BODIPY fluorophore at the meso position.^35^ Conjugation of AcroB “turns on” the fluorescence of the molecule, as loss of unsaturation at the BODIPY meso position removes the non-radiative relaxation pathway that otherwise renders AcroB non-fluorescent. The initial report detailing the development of AcroB focused on super-resolution localization of reactivity in mitochondria.^35^ In that study, gel experiments showed the vast majority of cellular fluorescent AcroB adducts were of low molecular weight, consistent with formation of AcroB-GSH adducts, positioning AcroB as a promising candidate to monitor cellular GSH-mediated LDE detoxification.

Initial solution-based experiments (Fig. S1A–C†) supported AcroB as a suitable candidate to monitor cellular LDE chemistry as AcroB conjugation with GSH was dependent on GSH concentration (Fig. S1A†), GST concentration (Fig. S1B†), and concentration of competing LDE (Fig. S1C†).

In HeLa cells, AcroB behaves as an LDE-mimic enabling observation of both cellular electrophile conjugation and electrophile-adduct export. Widefield fluorescence microscopy was chosen for cell imaging studies as this method enabled the simultaneous observation of cell fluorescence (AcroB conjugation and level of AcroB-adducts in the cell – AcroB retention) and background fluorescence (level of AcroB-adduct export – AcroB excretion, Fig. 1C). The exported adducts were confirmed as AcroB-GSH adducts by HPLC and mass spectrometry (Fig. S1G–I†). Movie M1† provides a view of a HeLa cell treated with 100 nM AcroB at 100× magnification and Movie M2† depicts a field of view (FOV) of HeLa cells at 20× magnification. At 100× magnification, subcellular AcroB-adduct movement was observed, while at 20× magnification, both cell fluorescence and background fluorescence increased over the 1 hour imaging window.

To quantify intracellular AcroB adducts and adduct export over time, we utilized the measurement of Corrected Total Cell Fluorescence (CTCF) and fluorescence background, respectively. CTCF represents the average total fluorescence per cell (and accounts for the detected background at each time point), while fluorescence background intensity represents the average intensity of each extracellular pixel (see Methods section in the ESI†). As fluorescence background intensity is dependent on the volume of media, imaging was performed with a uniform media volume to enable comparison of fluorescence background values across treatment conditions.

Analysis of 20× widefield imaging reveals that AcroB CTCF plateaued after ∼20 minutes while fluorescence background continued to increase (images presented as “Control – no wash” in top line of Fig. S1D† and quantification in Fig. S1F†). Removal of the extracellular pool of unreacted AcroB adducts through washing prevented further CTCF or background growth (images presented as “Control – wash” in middle line of Fig. S1D† and quantification in Fig. S1F†). These results are consistent with a steady state being reached during AcroB imaging between cellular import of unreacted AcroB, intracellular conjugation, and export. These observations established the response of AcroB to “control” conditions in HeLa cells, providing a baseline to characterize the response of AcroB to manipulation of the LDE detoxification pathway.

**Fig. 2:**
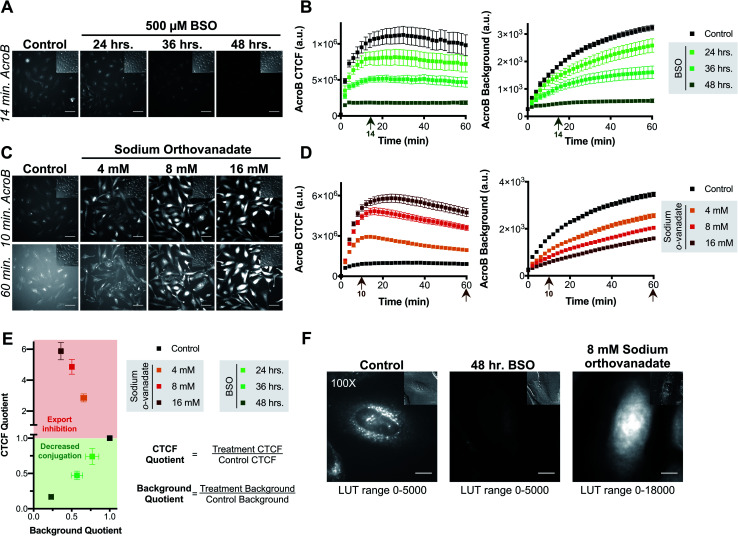
AcroB enables monitoring of cellular electrophile detoxification in live cells. (A and B) Inhibition of glutathione biosynthesis with BSO decreases conjugation of cellular electrophiles as monitored by both decreased AcroB cellular fluorescence and background fluorescence intensity. (A) Representative 20× widefield fluorescence and corresponding DIC (inset) images of HeLa cells with AcroB after no treatment (control) or treatment for 24–48 hours with 500 μM BSO. (B) Average AcroB corrected total cell fluorescence (CTCF) and fluorescence background curves representing *n* = 11 Fields of View (FOV, control), *n* = 6 FOV (24 h), *n* = 6 FOV (36 h) and *n* = 12 FOV (48 h) obtained from 20× magnification images. Arrow indicates 14 minute time point depicted in (A). (C and D) MRP-mediated electrophile adduct export inhibition with sodium orthovanadate decreases fluorescent AcroB-adduct export resulting in increased cellular fluorescence and decreased background fluorescence intensity. (C) Representative 20× widefield fluorescence and corresponding DIC (inset) images of HeLa cells with AcroB in the absence or presence of 4–16 mM sodium orthovanadate. (D) Average AcroB CTCF and fluorescence background curves representing *n* = 11 FOV (control), *n* = 11 FOV (4 mM), *n* = 12 FOV (8 mM) and *n* = 12 FOV (16 mM) obtained from 20× magnification images. Arrows indicate times depicted in (C). 20× imaging (A and C): images acquired over 60 minutes following AcroB addition, LUT range 0–6000, scale bar is 64 μm. All values (B and D) presented as mean ± SEM. (E) Electrophile detoxification Quotient (ElectrophileQ) plot constructed using values recorded 20 minutes after AcroB application from imaging conditions presented in panels (A–D). CTCF Quotient is the quotient of the average CTCF value for the indicated condition over that for control conditions. Background quotient is the analogous quotient determined from fluorescence background values. Control value is at 1.0–1.0 and is presented as a visual aid. The upper (red) region represents a regime where export inhibition dominates impairment of electrophile detoxification. The lower (green) region represents a regime where decreased reactivity dominates impairment of electrophile detoxification. (F) HeLa cell images acquired at 100× magnification after indicated treatment show high AcroB cytoplasmic fluorescence following export inhibition (right) and low overall fluorescence following decreased conjugation (middle). Images were acquired 30 minutes after AcroB application. The corresponding DIC images are shown (upper right inset). Scale bar is 12 μm. AcroB concentration = 100 nM for all experiments. *λ*_ex_ = 488 nm (0.1 mW for 20×, 0.05 mW for 100×).

Sensitivity of AcroB imaging to decreased cellular electrophile conjugation ability was demonstrated in studies following treatment with l-buthionine sulfoximine (BSO), a glutathione biosynthesis inhibitor (step 2, Fig. 1B).^36^ With increased length of BSO incubation (*i.e.*, decreased cellular GSH levels), both AcroB CTCF and background fluorescence decreased, consistent with reduced formation of AcroB adducts and an impaired cellular ability to handle electrophile stress following GSH depletion. See Fig. 2A for images of HeLa cells treated with AcroB after 24–48 hour incubation with 500 μM BSO and Fig. 2B for the quantification of AcroB CTCF and fluorescence background over 60 minutes following AcroB exposure. After 48 hour incubation with BSO, 100× imaging showed that the subcellular movement of AcroB punctate structures was retained, while the lower overall fluorescence intensity (both cellular and background) reflects the decreased level of AcroB conjugation (Fig. 2F – middle panel, Movie M3†).

The AcroB response to impaired electrophile adduct export was benchmarked using sodium orthovanadate, an MRP inhibitor that acts through blocking ATPase activity (step 3, Fig. 1B).^37,38^ Following treatment with increasing concentrations of sodium orthovanadate, AcroB adducts were retained in the cell, as shown by increased CTCF and decreased background levels. See Fig. 2C for images of HeLa cells treated with AcroB following incubation with 4–16 mM sodium orthovanadate and Fig. 2D for the quantification of AcroB CTCF and fluorescence background. The increased AcroB cell fluorescence following sodium orthovanadate treatment was located in the cytoplasm, as highlighted by 100× imaging (Fig. 2F – right panel, Movie M4†), consistent with observed retention of AcroB-GSH adducts formed in the cytoplasm that are otherwise exported in the absence of MRP inhibition. This AcroB-adduct retention was reversed and adduct export returned to control levels when sodium orthovanadate treatment was removed through washing and replacement of the sodium orthovanadate-containing imaging media with imaging media alone (sodium orthovanadate – wash condition) or containing AcroB (sodium orthovanadate – wash + replenish AcroB condition, Fig. S1E and F†).

Taken together, the results following BSO and sodium orthovanadate treatment demonstrate that AcroB has a response to LDE-adduct export impairment that is quantitatively and morphologically distinct from the response to decreased LDE conjugation ability.

The distinct responses of AcroB CTCF and background values to alterations in either LDE conjugation (Fig. 2A and B) or LDE-adduct export ability (Fig. 2C and D) were combined to generate the Electrophile detoxification Quotient assay (ElectrophileQ, see Fig. 2E), facilitating identification and mechanistic investigation of LDE detoxification impairment across cellular conditions, including ferroptosis. The ElectrophileQ plot presents the correlation between fluorescent AcroB cellular retention (CTCF quotient) and fluorescent AcroB excretion (background quotient) in a given cellular condition. To build the ElectrophileQ plot, CTCF and background fluorescence quotients were calculated by dividing the value obtained in a specific experimental condition (for example, 48 hour BSO incubation) by that recorded under the control conditions. The timepoint chosen for comparison of both CTCF and background parameters was 20 minutes after AcroB addition due to the steady state being reached in control conditions at approximately this time (Fig. S1F†). With increasing BSO incubation times, both CTCF and fluorescence background *decreased* relative to control conditions, establishing a standard curve for decreased conjugation alone and a regime (CTCF quotient < 1) where impaired GSH conjugation dominates LDE detoxification impairment (Fig. 2E, lower half of plot). Conversely, with increasing concentrations of sodium orthovanadate, CTCF *increased* and fluorescence background *decreased* relative to control conditions, establishing a standard curve for export inhibition alone and a regime (CTCF quotient > 1) where decreased export dominates LDE detoxification impairment (Fig. 2E, upper half of plot). Identification of detoxification impairment arising from mixed LDE conjugation and adduct export inhibition was demonstrated using ethacrynic acid (EA, Fig. S2†), a small molecule diuretic that has multiple effects on cellular electrophile detoxification including GSH depletion, GST inhibition and adduct export inhibition.^39–41^ Here, entry points in the ElectrophileQ plot fell between the two standard curves, and the analysis identified that while low concentrations of EA produced adduct export impairment alone, decreased GSH conjugation significantly contributed to detoxification impairment with increasing [EA] (Fig. S2E†).

The ability to deconvolute the effects a drug treatment has on both LDE conjugation and adduct export highlights the potential efficacy of ElectrophileQ for evaluating modulations to LDE detoxification capacity across a broad range of cellular conditions and allowed us to next mechanistically study LDE metabolism in ferroptosis.

### LDE detoxification impairment is a hallmark of ferroptosis

The role of LDE detoxification in ferroptosis was investigated using AcroB imaging and ElectrophileQ analysis in HT-1080 cells. Firstly, we standardized the ElectrophileQ plot for export inhibition with sodium orthovanadate in HT-1080 cells (Fig. S3A and B†). Additionally, we conducted complementary quantification of cellular electrophiles with Na-FA, a well-established aldehyde-reactive fluorogenic probe based on a hydrazine trap.^42–44^ To facilitate identification and mechanistic characterization of LDE detoxification ability during ferroptosis, two methods of ferroptosis induction were used, RSL3 and BSO,^11^ along with two modes of ferroptosis inhibition, RTA treatment (phenoxazine, PHOXN)^45^ and iron chelation (deferoxamine, DFO).^1^

**Fig. 3:**
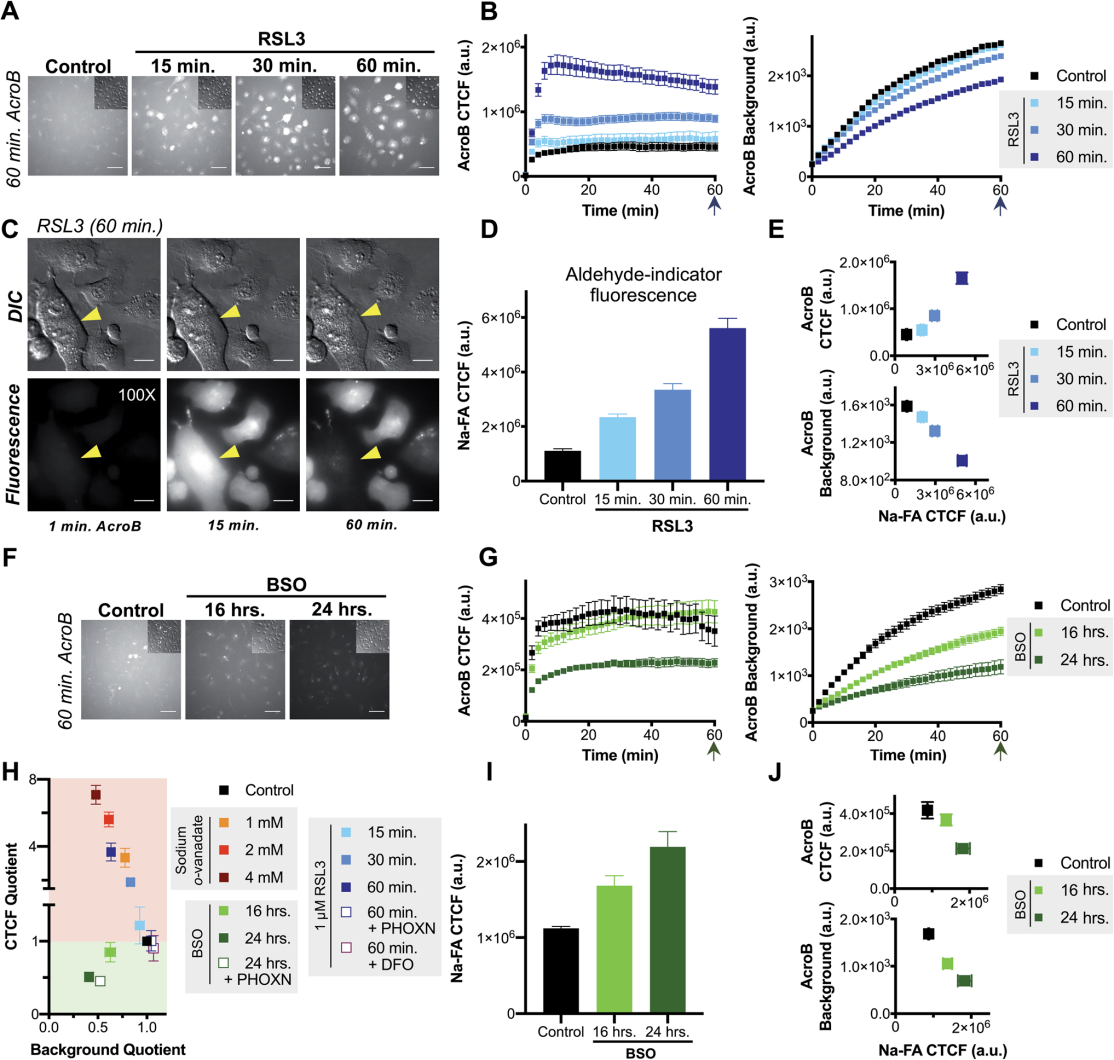
Ferroptosis is characterized by impaired electrophile detoxification and associated increased cellular electrophile levels. (A–E) RSL3-induced ferroptosis is marked by decreased electrophile adduct export and associated increased electrophile levels. (A) Representative 20× widefield AcroB fluorescence and corresponding DIC (inset) images of HT-1080 cells following 0–60 minute incubations with 1 μM RSL3. (B) Average AcroB CTCF and fluorescence background curves representing *n* = 15 FOV (control), *n* = 12 FOV (15 min) and *n* = 16 FOV (30 min, 60 min) obtained from 20× images. Arrow indicates 60 minute timepoint depicted in (A). (C) 100× imaging of HT-1080 cells following 60 minute treatment with 1 μM RSL3 shows large cytoplasmic fluorescence followed by loss of cell fluorescence in the emission channel and loss of pseudo three-dimensional relief shading in the DIC channel during cell membrane rupture. Fluorescence and corresponding DIC images presented. Scale bar is 12 μm. (D) Aldehyde probe Na-FA fluorescence levels (5 μM, 30 minute incubation) in HT-1080 cells after 0–60 minute incubations with 1 μM RSL3. Average of *n* = 8 FOV for all conditions. (E) AcroB CTCF or fluorescence background *vs.* Na-FA CTCF following RSL3 treatment. Values used are those calculated 30 minutes following dye treatment as shown in (B) and (D). (F–J) BSO-induced ferroptosis is marked by decreased electrophile conjugation and associated increased electrophile levels. (F) Representative 20× widefield AcroB fluorescence and corresponding DIC (inset) images of HT-1080 cells following 0–24 hour incubations with 500 μM BSO. (G) Average AcroB CTCF and fluorescence background curves representing *n* = 12 FOV (control), *n* = 16 FOV (16 h), *n* = 11 FOV (24 h) obtained from 20× images. Arrow indicates 60 minute timepoint depicted in (F). (H) ElectrophileQ plot constructed using values recorded 20 min after AcroB application for indicated conditions presented in (B), (G) and Fig. S3B and S4.† (I) Aldehyde probe Na-FA fluorescence levels (5 μM, 30 minute incubation) in HT-1080 cells after 0–24 h incubations with 500 μM BSO. Average of *n* = 16 FOV for all conditions. (J) AcroB CTCF or fluorescence background *vs.* Na-FA CTCF following BSO treatment. Values used are those calculated 30 minutes following dye treatment as shown in (G) or (I). AcroB concentration = 100 nM. *λ*_ex_ = 488 nm (0.1 mW for 20×, 0.05 mW for 100×). 20× imaging: images acquired over 60 minutes following AcroB addition, LUT range 0–4000, scale bar is 64 μm. All values presented as mean ± SEM.

Induction of ferroptosis with RSL3 in HT-1080 cells led to failure of electrophile detoxification through LDE-adduct export impairment. With increasing RSL3 incubation times, *i.e.*, during later stages of ferroptosis, we observed an increase in fluorescent AcroB adduct retention in HT-1080 cells. See Fig. 3A for 20× widefield images of HT-1080 cells incubated with 1 μM RSL3 for 15–60 minutes (additional timepoint images in Fig. S3D†) and Fig. 3B for the corresponding quantification showing that with longer RSL3 incubation, AcroB CTCF increased with a concomitant drop in background fluorescence. Analysis of the ElectrophileQ plot (Fig. 3H) revealed that the LDE detoxification impairment observed during RSL3-induced ferroptosis is fully accounted for by reduced LDE-adduct export alone, as the data points for RSL3 treatment fall in line with those for sodium orthovanadate treatment (Fig. S3A and B†). Fig. 3C and accompanying Movie M5,† conducted at 100× magnification (control presented in Fig. S3C†), highlight cytoplasmic retention of AcroB adducts during RSL3-induced ferroptosis (similar to sodium orthovanadate treatment – Movie M4†). Additionally, during the cell membrane rupture characteristic of ferroptosis (visualized in the DIC channel as a loss of pseudo three-dimensional relief shading), we recorded complete loss of the retained cytosolic AcroB adducts (yellow arrow in Fig. 3C, Movie M5, Fig. S3D†).

The LDE-adduct export impairment during RSL3-induced ferroptosis was associated with increased cellular electrophile accumulation. Aldehyde quantification with Na-FA (structure in Fig. S3E†) identified that increase in aldehyde signal was also dependent on the length of RSL3 exposure (Fig. 3D, see images in Fig. S3F†). A relationship between the length of ferroptosis induction, the level of electrophile-adduct export impairment and the amount of cellular aldehyde accumulation is shown in Fig. 3E, where both AcroB CTCF and AcroB background fluorescence are plotted against Na-FA relative aldehyde quantification. This result suggests that LDE-adduct export impairment during RSL3-induced ferroptosis increases electrophile accumulation, although this assay cannot distinguish between the intrinsic aldehyde level due to LOOH breakdown alone and the potential increase attributed to LDE-adduct export impairment. Treatment with ferroptosis inhibitors (PHOXN or DFO, Fig. S4A–F†) that prevent lipid autoxidation upstream of GPX4 inhibition led to complete recovery of basal LDE detoxification ability (Fig. 3H) and cellular aldehyde levels (Fig. S4D and F†).^1,45^

Taken together, the previous results suggest that the level of LDE production during RSL3-induced ferroptosis overwhelms cellular LDE-adduct export ability, leading to a roadblock in LDE detoxification and further LDE accumulation downstream of LOOH accumulation.

Induction of ferroptosis with BSO in HT-1080 cells altered cellular LDE handling through decreased electrophile conjugation ability and associated LDE accumulation. Fig. 3F depicts HT-1080 cells imaged 60 minutes following AcroB addition after ferroptosis induction with 500 μM BSO for 16–24 hours. Quantification of AcroB CTCF and background fluorescence (Fig. 3G) and ElectrophileQ analysis (Fig. 3H) confirmed that BSO treatment in HT-1080 cells led to LDE detoxification impairment through decreased cellular ability to form electrophile adducts. This result is consistent with the behaviour of BSO treatment in HeLa cells (Fig. 2). While the mechanism of LDE detoxification impairment during BSO-induced ferroptosis is distinct from that characterized during RSL3-induced ferroptosis (Fig. 3H), both ferroptosis inducers increased cellular aldehyde levels. An incubation-time dependent increase in Na-FA signal was recorded in HT-1080 cells following treatment with BSO (Fig. 3I, see images in Fig. S3G†). A relationship between the length of BSO incubation, the decrease in electrophile conjugation ability and the level of cellular aldehyde accumulation is established in Fig. 3J. Inhibition of lipid autoxidation with PHOXN during BSO treatment recovered control cellular electrophile levels (Na-FA imaging, Fig. S4G and J†) but LDE detoxification ability remained impaired relative to control conditions (Fig. 3H and S4G–I†). This result in is contrast with the complete recovery of control LDE metabolism observed following RSL3 treatment in the presence of PHOXN (Fig. S4A–D,† see above). However, the result is consistent with BSO treatment requiring lipid peroxidation to induce LOOH generation, aldehyde accumulation and ferroptosis, but BSO-induced inhibition of GSH biosynthesis being sufficient for gradual cellular GSH depletion and cellular sensitization to impaired electrophile detoxification. Similar experiments with DFO treatment for the extended imaging incubation times required for BSO treatment were not included as prolonged iron chelation prevented cell division.

AcroB imaging with ElectrophileQ analysis in combination with parallel cellular aldehyde quantification allowed us to identify and mechanistically characterize altered LDE metabolism in both RSL3 and BSO-induced ferroptosis, establishing LDE detoxification impairment associated with LDE accumulation as a hallmark of ferroptosis.

### LDE-adduct export impairment is linked to cell death in ferroptosis

**Fig. 4:**
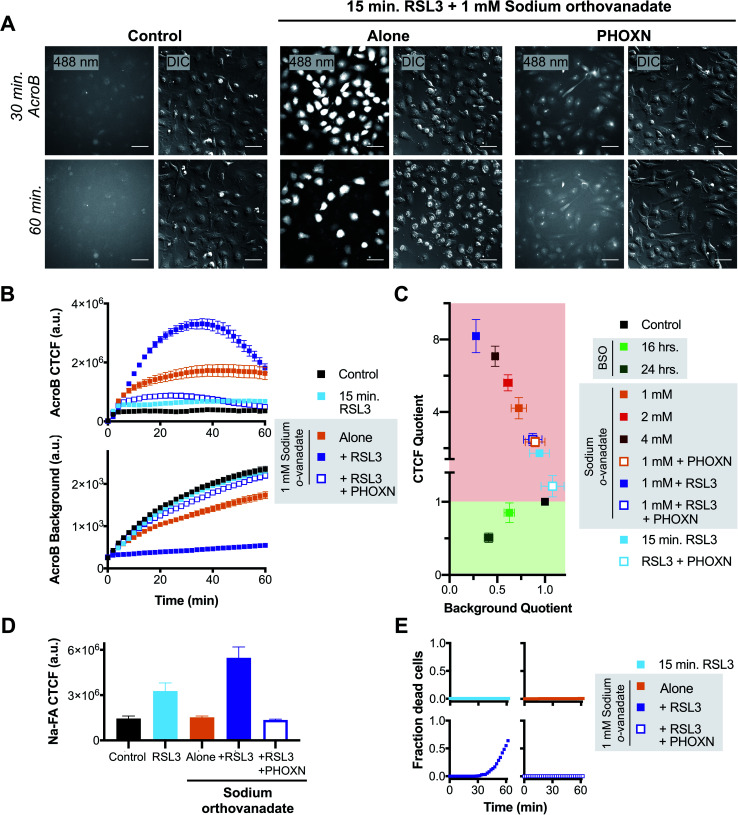
Exacerbated electrophile export impairment during ferroptosis induction increases aldehyde accumulation and cell death. (A) Representative 20× widefield AcroB fluorescence and corresponding DIC images of HT-1080 cells with/without treatments with 1 μM PHOXN (30 min incubation), 1 mM sodium orthovanadate (30 min incubation) and 1 μM RSL3 (15 min incubation). Images acquired over 60 minutes following AcroB addition, LUT range 0–4000, scale bar is 64 μm. (B) Average AcroB CTCF and fluorescence background curves representing *n* = 11 FOV (control), *n* = 9 FOV (15 min RSL3), *n* = 10 FOV (1 mM sodium orthovanadate), *n* = 9 FOV (sodium orthovanadate + RSL3) and *n* = 10 FOV (sodium orthovanadate + RSL3 + PHOXN) obtained from 20× widefield fluorescence images. AcroB concentration = 100 nM. (C) ElectrophileQ plot constructed using values recorded 20 min after AcroB application for indicated conditions presented in (C) and Fig. S5.† (D) Aldehyde probe Na-FA fluorescence levels (5 μM, 30 minute incubation) in HT-1080 cells after indicated treatments. Average of *n* = 5 FOV for all conditions. (E) Fraction of dead cells as determined by 20× widefield propidium iodide (10 μM) fluorescence imaging for indicated conditions. All values presented as mean ± SEM. *λ*_ex_ = 488 nm (0.1 mW).

We next sought to test whether the LDE detoxification impairment that occurs during ferroptosis is associated to eventual ferroptotic cell death. We thus performed AcroB and ElectrophileQ-based imaging studies where a short (15 min) and sub-lethal incubation with RSL3 was either combined with mild LDE-adduct export impairment (1 mM sodium orthovanadate) alone or with sodium orthovanadate in the presence of PHOXN to prevent lipid peroxidation (see Fig. 4 and additional controls presented in Fig. S5†). Pre-sensitization of HT-1080 cells with sodium orthovanadate treatment followed by short incubation with RSL3 led to a large increase in LDE-adduct export inhibition. See Fig. 4A for 20× widefield images of the combined sodium orthovanadate and RSL3 treatment (middle panel, see Fig. S5A† for additional control images), Fig. 4B for quantification of AcroB CTCF and background (see Fig. S5C† for additional controls) and Fig. 4C for ElectrophileQ analysis. The initial increase in AcroB CTCF and decrease in background fluorescence following the combined RSL3 and sodium orthovanadate treatment greatly exceeded those recorded with either 1 mM sodium orthovanadate or 15 min RSL3 incubation alone (Fig. 4B and C). The observed decrease in AcroB CTCF value of this combined treatment after ∼40 minutes of imaging corresponds to widespread cell rupture and release of the fluorescent AcroB-adducts. These experiments highlight that the combined RSL3 and sodium orthovanadate treatment results in a synergistic impairment of LDE export.

Relative aldehyde quantification with Na-FA imaging following combined 15 min RSL3 and 1 mM sodium orthovanadate treatment confirmed a concomitant increase in cellular aldehyde levels (Fig. 4D, S5B and D†). For the combined treatment, the Na-FA aldehyde fluorescence level recorded was comparable to that observed following 60 minute RSL3 incubation without sodium orthovanadate (Fig. 3D).

Addition of PHOXN to the combined sodium orthovanadate and RSL3 treatment aborted the synergistic impairment of LDE detoxification, restoring the ElectrophileQ values to those recorded with sodium orthovanadate and PHOXN treatment (Fig. 4A and C). Addition of PHOXN to the combined treatment also recovered the level of cellular aldehydes, as recorded with Na-FA, to the value measured during both control conditions and sodium orthovanadate treatment without RSL3 (Fig. 4D). These results with PHOXN treatment indicate that the LDE detoxification impairment and aldehyde accumulation during combined mild sodium orthovanadate and RSL3 treatment are dependent on lipid peroxidation, consistent with that observed during RSL3 treatment alone (Fig. 3A–E and S4A–D†). This suggests that sodium orthovanadate merely accelerates the LDE detoxification failure already characteristic of late-stage RSL3-induced ferroptosis.

Most importantly, imaging with propidium iodide to assess cell viability showed that cell death occurred only during the combination treatment of 1 mM sodium orthovanadate and 15 min RSL3 (see Fig. 4E and S5E†). No cell death was observed during either mild sodium orthovanadate or short RSL3 exposure, or in conditions where PHOXN pre-treatment occurred (see panels in Fig. 4E). Comparison of the DIC *vs.* fluorescence images in the middle panel in Fig. 4A highlights the loss of AcroB cell fluorescence concomitant to the loss of pseudo three-dimensional relief shading in the DIC channel during the high incidence of cell rupture that occurs following the combined treatment. This observed loss of membrane integrity is consistent with the loss of AcroB adducts and DIC contrast during ferroptotic cell rupture following 60 minute RSL3 treatment (Fig. 3C, Movie M5†).

**Scheme 1:**
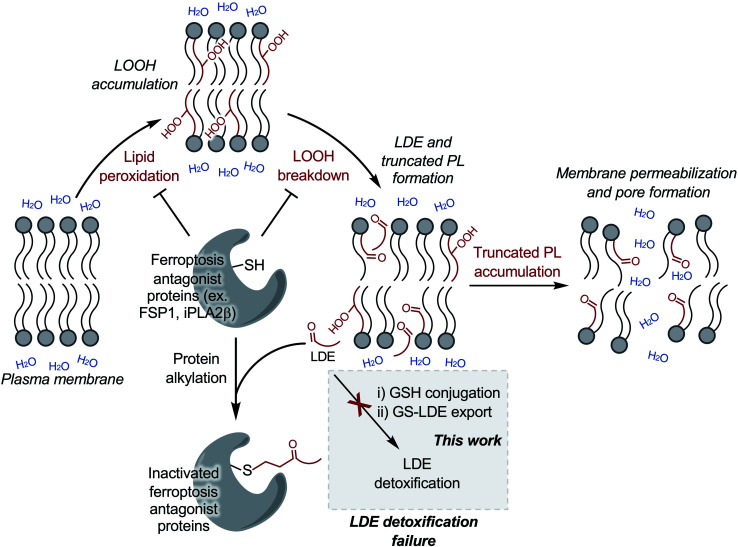
LDE detoxification failure is a critical stage of ferroptotic cell death. Putative pathway linking LOOH accumulation in ferroptosis to LDE/truncated PL accumulation and to membrane permeabilization. Induction of ferroptosis leads to LOOH accumulation *via* lipid peroxidation. LOOH breakdown produces downstream LDE and truncated PL formation. Increased LDE production overwhelms or inhibits the two-step LDE detoxification pathway, leading to amplified cellular LDE accumulation. Several critical ferroptosis antagonist regulatory proteins including FSP1 and iPLA2β are susceptible to alkylation. Putative inactivation of FSP1 and iPLA2β, among other proteins, following LDE alkylation would remove inhibition of lipid peroxidation and increase retention of membrane LOOHs, respectively, leading to increased LOOH breakdown products in a positive feedback loop. Eventually, the level of truncated PLs reaches a critical concentration leading to membrane permeabilization.

Taken together, these results support a link between LDE detoxification failure and cell death *via* membrane rupture (Scheme 1). While treatment with the ATPase inhibitor sodium orthovanadate may additionally interfere with cell metabolism through undesired off target effects, our observations demonstrate a clear relationship between decreased ability to detoxify LDEs (Fig. 4A–C), elevated levels of cellular electrophiles (Fig. 4D) and increased ferroptosis susceptibility (Fig. 4E). This relationship suggests that inability to detoxify LDEs may have pathological cellular consequences during ferroptosis.

## Discussion

To investigate the role of LDE metabolism in ferroptosis, we developed a methodology (ElectrophileQ) for the real-time monitoring of LDE detoxification in live cells (Fig. 2). Correlating the cellular retention *vs.* excretion of the fluorogenic lipophilic electrophile AcroB facilitated monitoring of both the critical GSH conjugation and adduct export steps of the LDE detoxification pathway. The ability to simultaneously monitor both stages of this pathway enables not only the identification of LDE detoxification impairment, but also mechanistic analysis of the mode of inhibition (demonstrated with ethacrynic acid, Fig. S2†).

The application of AcroB imaging with ElectrophileQ analysis to study ferroptosis revealed that following lipid peroxidation and LOOH accumulation, LOOH breakdown to electrophiles (LDEs and electrophilic truncated PLs) occurs associated with impaired LDE detoxification (Fig. 3). Our investigation reveals this impaired detoxification takes one of two forms. During RSL3-induced ferroptosis, LDE-adduct export is inhibited, consistent with insufficient MRP-mediated export of cellular GSH-electrophile adducts (Fig. 3A–E). Here, inhibition is due to either the level of LDE production overwhelming the available MRP export channels (as GSH conjugation level remains unimpaired, Fig. 3H), or the inhibition of MRP channels by specific GS-LDE adducts.^46^ In contrast, upon induction of ferroptosis with BSO, impaired detoxification arises from a depleted level of cellular GSH leading to reduced/impaired GSH-mediated LDE conjugation (Fig. 3F–J). Our observation that GSH depletion alone (following BSO treatment) leads to LDE detoxification impairment and LDE accumulation without experimental manipulation of MRP activity emphasizes the requirement of cellular GSH for protection against LDEs generated during ferroptosis. This relationship between GSH level and cell survival is consistent with previous work demonstrating that GSH efflux prior to ferroptosis induction increased ferroptosis susceptibility^47^ – once the cellular GSH pool is consumed, the number of available export channels is irrelevant as both GPX4 activity and LDE conjugation are inhibited leading to increased LDE accumulation.

Crucially, exacerbation of LDE-adduct export impairment during ferroptosis *via* MRP inhibition with sodium orthovanadate was associated with increased cellular ferroptosis susceptibility (Fig. 4). This finding improves molecular understanding of ferroptotic cell death downstream of LOOH accumulation as it positions GSH-conjugation combined with MRP-mediated LDE-adduct export as a potential defence mechanism used by the cell to prevent or delay cell death following ferroptosis induction and suggests that LDE detoxification failure is a precursor to ferroptotic cell death (Scheme 1).

While increased production of LDEs during ferroptosis is expected following breakdown of the accumulated cellular LOOHs, surprisingly, we demonstrate that this increased LDE production brings about a failure of LDE detoxification that ultimately further amplifies the cellular electrophile load (Fig. 3 and 4). In this regard, it has been previously shown that high LDE levels lead to widespread protein alkylation during ferroptosis.^23^ Importantly, these alkylated proteins likely include key ferroptosis regulatory proteins bearing reactive cysteine residues. Multiple ferroptosis regulatory proteins are susceptible to LDE alkylation including VDAC2 (ref. 23) and FSP1,^48–50^ while others are known to be covalently deactivated by electrophiles (for example iPLA2β).^26,51,52^ We postulate that inactivation of proteins acting as ferroptosis antagonists (for example FSP1 and iPLA2β) following LDE detoxification impairment exacerbates LOOH accumulation and breakdown further, creating a positive feedback loop that places LDE detoxification impairment and amplified electrophile accumulation as a potential point of no return during ferroptotic cell death (Scheme 1). This proposed mechanism identifies the LDE detoxification pathway as a potential ferroptosis-relevant therapeutic target, where inhibition would increase ferroptosis susceptibility (*e.g.* cancer therapy) and activation would aid in prevention of ferroptotic cell death (*e.g.* degenerative disease management).

The truncated PLs produced alongside LDEs during LOOH breakdown are also expected to play a critical role in ferroptosis. Consistent with the pore formation observed in ferroptosis,^17,18^ truncated PLs have been shown both computationally^53–55^ and in model membranes^55–58^ to induce membrane disruption, thinning, and pore formation. At the time of submission of this work we became aware of a recent study by Friedmann Angeli *et al.* showing that the specific formation of short chain truncated PLs is responsible for membrane permeabilization in ferroptosis.^22^

LDE detoxification failure and the ensuing alkylation of critical ferroptosis regulatory proteins together with membrane permeabilization by truncated PLs provides a putative molecular cell death mechanism of ferroptosis (Scheme 1). Whether specific LDEs are responsible for either MRP inhibition or the alkylation of critical ferroptosis regulatory proteins remains to be discovered.

## Conclusion

Detoxification of LDEs is critical for the maintenance of cellular health as these highly reactive downstream products of lipid peroxidation are capable of protein alkylation and inactivation. The known formation of LDEs during ferroptosis inspired us to develop a method to observe LDE detoxification in live cells. Here, fluorogenic electrophile (AcroB) imaging in combination with ElectrophileQ analysis enabled monitoring of both the GSH conjugation and MRP-mediated adduct exports steps of the LDE detoxification pathway. This strategy allowed us to identify LDE detoxification impairment as a hallmark of ferroptosis downstream of LOOH accumulation and to propose failure of LDE detoxification as a critical stage of ferroptotic cell death. LDE detoxification impairment, shown to occur either through decreased conjugation (following GSH depletion with BSO treatment) or through inhibition of LDE-adduct export (following GPX4 inhibition by RSL3) ultimately amplifies the level of cellular electrophiles. Our work supports a putative ferroptotic cell death mechanism where LDE detoxification failure initiates a positive feedback loop resulting in increased LDE and truncated PL levels and eventual cell death *via* membrane permeabilization. In general, we envision AcroB imaging with ElectrophileQ analysis becoming an invaluable addition to the chemical toolbox for studying ferroptosis and cellular health, not only providing a method to screen for inhibitors or regulators of ferroptosis that act downstream of LOOH accumulation, but also a platform to study secondary cellular consequences of small molecule drugs and electrophilic drug metabolites.

## Data availability

All experimental data is available within the article and the ESI.†

## Author contributions

A. T. M. V. K.: conceptualization, methodology, formal analysis, investigation, writing – original draft, writing – review and editing, visualization. R. K.: software, investigation. G. C.: conceptualization, methodology, resources, writing – review and editing, supervision, project administration, funding acquisition.

## Conflicts of interest

The authors declare no conflicts of interest.

## Supplementary Material

SC-013-D2SC00525E-s001

SC-013-D2SC00525E-s002

SC-013-D2SC00525E-s003

SC-013-D2SC00525E-s004

SC-013-D2SC00525E-s005

SC-013-D2SC00525E-s006
