# Supplementary material for: Live-cell imaging reveals impaired detoxification of lipid-derived electrophiles is a hallmark of ferroptosis
Source: Chem Sci. 2022 Aug 1;13(33):9727–38. doi: 10.1039/d2sc00525e (PMC9400630; doi:10.1039/d2sc00525e)
Supplement: SC-013-D2SC00525E-s001 [file SC-013-D2SC00525E-s001.pdf]

Electronic Supplementary Material (ESI) for Chemical Science.
This journal is © The Royal Society of Chemistry 2022

Supplementary Information

Live-cell imaging reveals impaired detoxification of lipid-derived electrophiles is a hallmark of ferroptosis

*Antonius T. M. Van Kessel, Ryan Karimi and Gonzalo Cosa**

[1]Department of Chemistry, McGill University, 801 Sherbrooke Street West, Montreal, Quebec H3A 0B8, Canada.

*To whom correspondence should be addressed: Gonzalo Cosa

Email: gonzalo.cosa@mcgill.ca

Contents

- Materials and Methods (Page S3)
  - Materials (Page S3)
  - Microplate Assays (Page S3)
    - Measuring AcroB reactivity with GSH and GST (Page S3)
    - Measuring AcroB reactivity with GSH and GST in the presence of trans-2-decenal (Page S3)
  - Cell Culture (Page S3)
  - Imaging (Page S3)
    - Widefield microscopy (Page S3)
    - General imaging conditions (Page S4)
    - Sodium orthovanadate imaging (Page S4)
    - L-buthionine-sulfoximine imaging (Page S5)
    - Ethacrynic acid imaging (Page S5)
    - RSL3 imaging (Page S5)
    - Phenoxazine or DFO imaging (Page S5)
  - Image Analysis (Page S6)
    - Widefield fluorescence values quantification (Page S6)
    - ElectrophileQ assay analysis (Page S6)
  - Identification of AcroB adduct exported from live cells (Page S6)

- Supplemental Figures (Page S8)
  - **Figure S1.** AcroB enables the observation of both stages of LDE detoxification – intracellular GSH conjugation and adduct export (Page S8-S9)
  - **Figure S2.** AcroB distinguishes between different modes of electrophile detoxification impairment following ethacrynic acid treatment (Page S10)
  - **Figure S3.** Supplemental images and data related to Figure 3: Ferroptosis is characterized by impaired electrophile detoxification and associated increased electrophile levels. (Page S11)
  - **Figure S4.** Treatment with ferroptosis inhibitors recovers LDE metabolism following ferroptosis induction (Page S13)
  - **Figure S5.** Sodium orthovanadate pre-treatment leads to exacerbated electrophile export impairment, aldehyde accumulation and cell death following sub-lethal RSL3 treatment that is prevented with phenoxazine (Page S15)

- Supplemental Movie Legends (Page S16)

- References (Page S17)

Materials and Methods

Materials. HPLC-grade solvents, buffers, imaging media, growth media and all other reagents for cell culture were purchased through ThermoFisher Scientific. AcroB and Na-FA were synthesized according to previously reported procedures.[1, 2] All other chemicals were purchased from Sigma-Aldrich, Co. and used without further purification.

Microplate Assays. Microplate assays were measured on a Biotek Synergy 2 multimode microplate reader.

Measuring AcroB reactivity with GSH and GST. 10 mM GSH stock solution was prepared by dissolving 30.7 mg GSH in 10 mL PBS. 1 mg/mL GST from equine liver stock solution was prepared by dissolving 1.0 mg of GST from equine liver in 1 mL PBS. 400 µM AcroB stock was prepared in acetonitrile. Wells on the microplate were prepared to contain 0-200 µM GSH (30 µL a dilution series prepared from the 10 mM stock), 1 µM AcroB (50 µL of 6 µM AcroB in PBS prepared from the 400 µM stock), and 0-270 µg/mL GST from equine liver (8.11 µL from a dilution series prepared from the 1.0 mg/mL stock). Well volume of 300 µL was achieved through addition of the requisite volume of PBS. Microplate (black, flat clear bottom) was incubated at 37°C and the fluorescence reading at 520 nm was recorded every 10 minutes for 3 hours upon excitation at 475 nm with a sensitivity parameter of 100.

Measuring AcroB reactivity with GSH and GST in the presence of trans-2-decenal. GSH, GST from equine liver and AcroB stock solutions prepared as detailed above. 64 mM trans-2-decenal stock solution was prepared by diluting 11.7 µL trans-2-decenal in 1 mL DMSO. Wells on the microplate were prepared to contain 100 µM GSH (30 µL of 1 mM GSH prepared from the 10 mM stock), 1 µM AcroB (50 µL of 6 µM AcroB in PBS prepared from the 400 µM stock), 27 µg/mL GST from equine liver (8.11 µL of the 1 mg/mL stock) and 0-64 µM trans-2-decenal. Well volume of 300 µL was achieved through addition of the requisite volume of PBS. Microplate (black, flat clear bottom) was incubated at 37°C and the fluorescence reading at 520 nm was recorded every 10 minutes for 3 hours upon excitation at 475 nm with a sensitivity parameter of 100.

Cell Culture. HeLa cells (ATCC CCL-2) and HT-1080 cells (CCL-121) were cultured in Dulbecco's modified Eagle's medium (DMEM) containing high glucose, L-glutamine, phenol red, and sodium pyruvate (Gibco), supplemented with 10% fetal bovine serum (FBS, Gibco), 1% penicillin-streptomycin and, for HT-1080 cells, 1% non-essential amino acids (NEAA, Gibco) – hereafter "DMEM" refers to DMEM including the indicated growth supplements. Cells were maintained at 37°C (5% CO_2) in a humidified atmosphere. Cells were trypsinized and split 1/10 (HeLa) or 1/20 biweekly (HT-1080) when 90% confluency was reached. Cells were passaged a maximum of 30 times and monitored for mycoplasma infection.

Imaging.
Widefield microscopy. Widefield fluorescence and differential interference contrast (DIC) imaging were performed using a widefield objective-based setup consisting of an inverted microscope (Nikon Eclipse Ti) equipped with a Perfect Focus System (PFS) with either a 100× oil-immersion objective (Nikon CFI SR Apochromat TIRF 100×, NA = 1.49) or a 20× air objective (Nikon CFI Plan Apo VC 20× objective, NA = 0.75). Cells were maintained at 37°C and 5% CO_2 in a

humidified atmosphere using a stage-top incubator (Tokai Hit). 488 nm laser excitation was used at powers of 0.1 mW for 20× and 0.05 mW for 100× imaging measured out of the objective; the beam was coupled into the microscope objective using a multiband beam splitter (ZT488/640rpc, Chroma Technology). Emission was spectrally filtered with a ZET488/640m emission filter (Chroma Technology). A motorized filter block turret was used for multichannel imaging to change between the 488 and DIC channels to enable a corresponding DIC image with each widefield fluorescence imaging. Fluorescence emission was collected though the same objective and captured on a back illuminated electron multiplying charge coupled device (EM-CCD) camera (Andor iXon Ultra DU-897). Optical configuration controlled through Nikon NIS-Elements software: auto exposure (300 ms), readout mode (EM Gain 1 MHz at 16-bit), EM gain multiplier (17), conversion gain (Gain 3).

General imaging conditions. Unless otherwise indicated, cells were plated 24 hours prior to microscope imaging on a 35 mm glass imaging dish (FluoroDish, World Precision Instruments, Inc.) pre-coated with fibronectin (1 µg/cm^2 – for 100× imaging), on an 8-well ibidi-Treat μ-slide (for HeLa 20× imaging) or on fibronectin coated 8-well glass-bottomed ibidi μ-slide (for HT-1080 20× imaging) in DMEM containing growth factors. In order to reach approximately 50% confluency during imaging, 50,000 trypsinized cells suspended in 1 mL DMEM were plated on FluoroDishes and 15,000 trypsinized cells suspended in 300 µL DMEM were plated in each well of the 8-well μ-slides. For imaging, the growth media was removed and replaced with Live Cell Imaging Solution (LCIS, ThermoFisher) – LCIS supplemented with 5 mM glucose (LCIS + glucose) for HT-1080 cells. More specific imaging details for each treatment are below.

After 24-hour incubation on the imaging dish, cell media was removed and cells were washed 3 times with LCIS, and 1 mL (FluoroDish) or 200 µL (8-well μ-slide) LCIS was left on the cells (LCIS + glucose for HT-1080 cells). Once loaded onto the microscope, cell fields of view (FOV) for imaging were located and their coordinates saved on the PFS; 1 FOV was used for 100× imaging on the FluoroDish, while 2 FOV were chosen in each of the 8 wells on the ibidi μ-slide (16 FOV per imaging dish, see below).

Dye solutions were prepared from 30 µM (AcroB), 1.5 mM (Na-FA) or 3 mM (Propidium iodide) stock solutions in DMSO. 5 µL of the respective dye stock was added to 495 µL LCIS (LCIS + glucose for HT-1080 cells) in order to obtain a stock solution of 300 nM (AcroB), 5 µM (Na-FA) or 10 µM (Propidium iodide) and a final percentage DMSO on the cells of 0.33%. Immediately before imaging, dye was added as the 300 nM stock solution in LCIS to reach a final concentration in the imaging media of 100 nM (500 µL added to the FluoroDish, 100 µL added to 8-well ibidi μ-slide). The volume of imaging media was set to a uniform 300 µL for ibidi μ-slide experiments as fluorescence background quantification is dependent on the volume of extracellular media; the use of the humidified incubator for imaging also ensures minimal evaporation of imaging media. For 20× widefield imaging, each FOV was imaged every 2 minutes for 60 minutes (for AcroB and Propodium iodide) or every 10 minutes for 30 minutes (for Na-FA). For 100× widefield AcroB imaging, cells were imaged on both the DIC and 488 nm fluorescence channels every 30 seconds for 30 minutes. Any deviations from this protocol during the treatments are indicated below.

Sodium orthovanadate imaging. Stock solution of sodium orthovanadate[3] was made by dissolving 367.8 mg sodium orthovanadate in 10 mL MilliQ water. pH was adjusted to pH 10.0 using 1 M

HCl (solution turns yellow). After 10 minutes of heating the solution in boiling water, solution became colourless once more and pH was readjusted to 10.0 using 1 M HCl and 1 M NaOH. As solution did not return to a yellow colour, this process was not repeated. Sodium orthovanadate concentration was measured to be 0.137 M using the extinction coefficient at 265 nm of 2925 $M^{-1}cm^{-1}$. Aliquots of this solution were stored at -20°C, and diluted with media (DMEM, DMEM + NEAA, LCIS or LCIS + glucose) on the day of imaging to obtain 1-16 mM solutions. Media exchange was performed into DMEM (HeLa cells) or DMEM + NEAA (HT-1080 cells) containing the requisite [sodium orthovanadate] for a 30-minute (HT-1080) or 60-minute (HeLa) incubation at 37°C. Parallel control conditions consisted of a 30 or 60-minute incubation in media alone. Imaging performed in LCIS or LCIS + glucose containing the requisite [sodium orthovanadate] and pre-imaging LCIS washing also performed with sodium orthovanadate solutions. Wash conditions involved removing the imaging media after 16 minutes of imaging with AcroB (8 images taken per FOV) and replacement with the indicated solution (300 µL LCIS or 300 µL LCIS containing 100 nM AcroB).

L-buthionine-sulfoximine imaging. In order to facilitate 48-hour incubations with L-buthionine-sulfoximine (BSO) in HeLa cells, cells were plated to ¼ the normal density on imaging dishes 72 hours prior to imaging. In order to facilitate 24-hour incubations with BSO in HT-1080 cells, cells were plated to 1/3 the normal density on imaging dishes 48 hours prior to imaging. 50 mM BSO stock solutions in PBS were filtered and a 10-fold dilution was performed to obtain 5 mM BSO in DMEM or DMEM + NEAA. Cell media was spiked with BSO to a final concentration of 500 µM BSO at the reported time prior to imaging (16, 24, 36 or 48 hour incubation). Parallel control conditions replicated the 48 (HT-1080) 72-hour (HeLa) plating time.

Ethacrynic acid imaging. 33 mM stock solution of ethacrynic acid in DMSO was used and diluted into DMEM or LCIS to obtain working solutions of 10, 20 and 40 µM ethacrynic acid. 2 hours prior to imaging, a media exchange was performed on HeLa cells into the respective ethacrynic acid in DMEM solution, and cells were incubated at 37°C for 2 hours. After washing cells 3 times with LCIS (*vide supra*), 200 µL LCIS containing the respective ethacrynic acid concentration was added onto the cells. Dye solutions were also made with LCIS containing 10, 20 or 40 µM ethacrynic acid. Parallel control conditions consisted of a media exchange into fresh DMEM for a 2-hour incubation.

RSL3 imaging. 300 µM stock solution of RSL3 in DMSO was used and 16.6 µL was diluted into 484 µL DMEM + NEAA to obtain a working solution of 10 µM RSL3. 30 minutes prior to imaging, 1/10 dilution onto HT-1080 cells was performed for the 15-60 minute incubation with 1 µM RSL3 at 37°C. RSL3 was not present in the LCIS + glucose imaging media following washing. For conditions combining sodium orthovanadate and RSL3 treatments, sodium orthovanadate treatment occurred 30 minutes prior to spiking the media with RSL3 for the 15-minute RSL3 incubation. For conditions combining sodium orthovanadate, RSL3 and PHOXN treatments, PHOXN treatment occurred 30 minutes prior to sodium orthovanadate treatment.

Phenoxazine or DFO imaging. For phenoxazine (PHOXN) treatment, a 10 mg/mL stock solution of PHOXN was prepared in DMSO and diluted into DMEM + NEAA to a final concentration of 3 µM PHOXN. A 1/3 dilution was performed onto cells to reach a final [PHOXN] of 1 µM for a 30-minute pre-incubation prior to any additional treatments. For DFO treatment, a 10 mg/mL stock

solution was prepared in PBS and diluted into DMEM + NEAA to a final concentration of 300 μM DFO. A 1/3 dilution was performed onto cells to reach a final [PHOXN] of 300 μM for a 60-minute pre-incubation prior to any additional treatments.

Image Analysis.
All images were processed using the FIJI imaging processing package.[4]

Widefield fluorescence values quantification. Corrected total cell fluorescence (CTCF) and background fluorescence values were quantified adapting a previously published protocol.[5] Equation S3 was used to calculate CTCF:

$$\mathrm{CTCF} = \frac{\mathrm{IntDensity}_{\mathrm{totalcellarea}} - (\mathrm{total\ cell\ area} \times \mathrm{BkgFluorescence})}{N_{\mathrm{cells}}} \quad \mathrm{(S3)}$$

where $\mathrm{IntDensity}_{\mathrm{totalcellarea}}$ is the integrated intensity for the pixels determined to contain cells, total cell area is the number of pixels containing cells, BkgFluorescence is the mean fluorescence intensity per pixel for all pixels not containing cells and N_{cells} is the manually counted number of cells in the image. CTCF measurements and errors presented are the average and standard error of the mean from multiple fields of view (number indicated where corresponding data is presented). Background fluorescence as reported corresponds to BkgFluorescence (ie. a per pixel value).

In order to identify the area of the image containing cells, an ImageJ macro was used to analyze the corresponding DIC image. Initially, a variance filter with a radius of 4 pixels was applied to the DIC image, where areas of high variance represent the presence of a cell. A binary image was generated, and the Fill Holes function was applied. The Analyze Particles function was then used to fill any remaining holes smallers than 20 pixels^2 in the binary image, setting their pixel value to 255 and a dilate function was applied. The binary image was used to create a selected area containing cells, and the area without cells. Total cell area and total background area are measured from this binary image for each frame, facilitating the measurement of $\mathrm{IntDensity}_{\mathrm{totalcellarea}}$ and BkgFluorescence.

ElectrophileQ assay analysis. All values for the ElecrophileQ plots are calculated using CTCF and background fluorescence intensity values obtained from quantification of 20x widefield imaging experiments. Values chosen for comparison are those calculated from the images taken 20 minutes after AcroB application. CTCF quotient is calculated by taking the quotient of the average CTCF from the given treatment condition and the average control CTCF value. Background fluorescence quotient is calculated by taking the quotient of the average fluorescence background from the given treatment condition and the average control fluorescence background value. Due to the use of widefield illumination, photobleaching level is assumed to be uniform between the cell and background regions and is therefore not accounted for in the analysis.

Identification of AcroB adduct exported from live cells. A 90% confluent 10 cm dish of HT-1080 cells was washed with LCIS and treated with 3 mL LCIS containing 1 μM AcroB. Following 1-hour incubation at 37°C, incubation media was removed and filtered. HPLC purification carried out on an Agilent Technologies Infinity II equipped with an InfinityLab Poroshell 120 Phenyl-Hexyl column, dimensions are 4.6 x 150 mm. Particle size is 2.7 μm, pore size is 120 angstroms.

Elution achieved using a gradient of 100% water to 50:50 water:acetonitrile over 4 minutes, followed by 4 minutes of 50:50 water:acetonitrile, injection volume = 20 µL. AcroB adduct identified by absorbance at 503 nm and fluorescence at 520 nm (excitation 485 nm). Fraction collection gated by fluorescence detection at 520 nm (excitation 485 nm). Mass spectrometry identification of adduct identity performed on a Bruker maXis impact. Injection via LC, detection channel absorbance at 503 nm. ESI positive ion mode, HR accurate mass obtained, sodium formate calibration. Molecular formula assignment achieved using Bruker Compass DataAnalysis 4.2.

Supplemental Figures

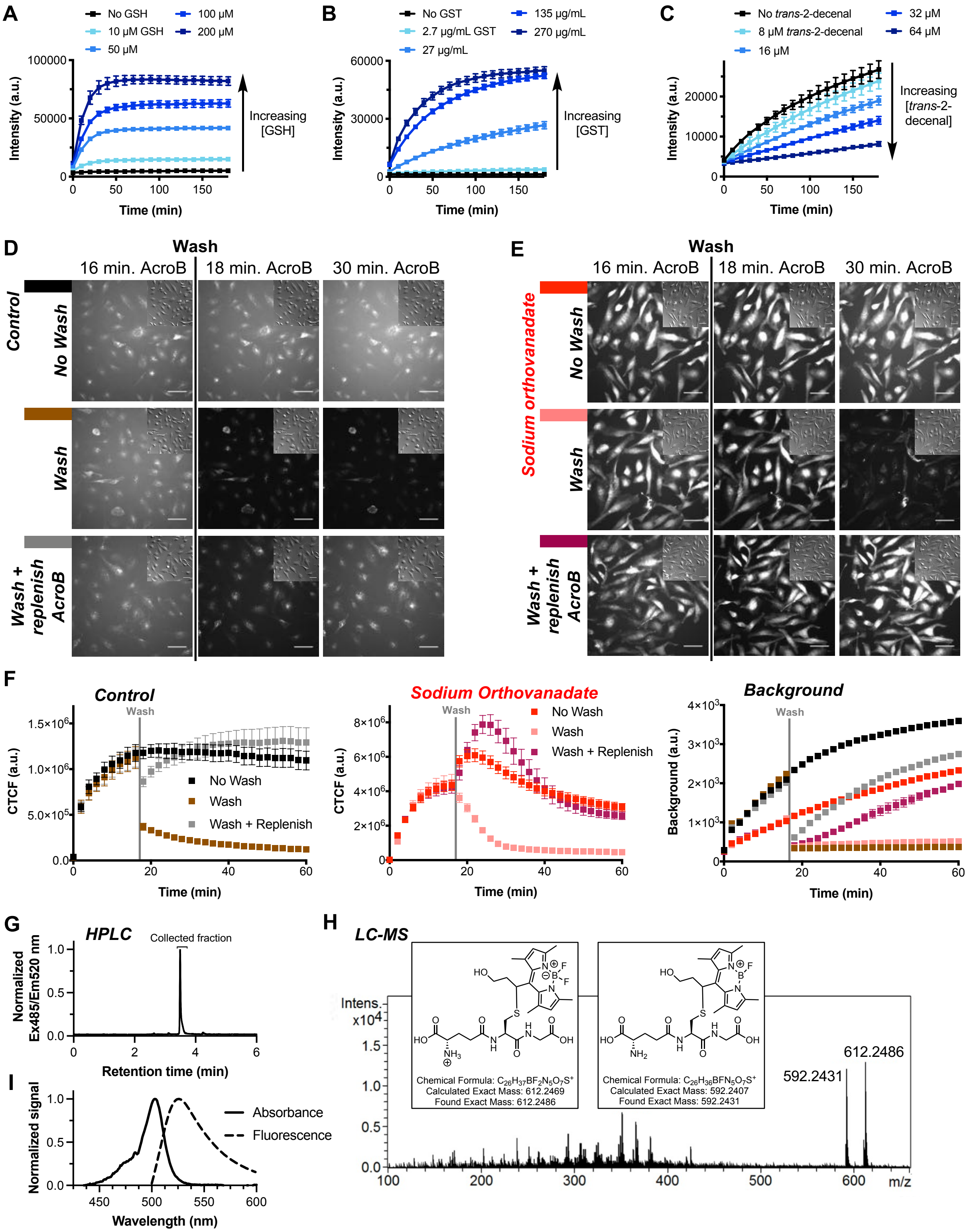

Figure S1. AcroB enables the observation of both stages of LDE detoxification – intracellular GSH conjugation and adduct export. (A-C) Rate of AcroB reaction in solution as a function of increasing concentrations of GSH, GST and competing electrophiles. Fluorescence enhancement of AcroB (1 μM) in the presence of (A) 27 µg/mL GST and varying [GSH] (0-200 µM, as indicated), or (B) 100 µM GSH and varying [GST] (0-270 µg/mL, as indicated), or (C) 100 µM GSH, 27 µg/mL GST and varying [*trans*-2-decenal] (0-64 µM, as indicated). λ_{ex} = 475 nm, λ_{em} = 520 nm. Values presented as mean ± SEM. Difference in absolute values across panels is due to experiments being performed with different lots of purchased GST from equine liver. (D-F) Washing of HeLa cells after AcroB application and sodium orthovanadate treatment shows continual influx of unreacted AcroB drives cell fluorescence response and removal of sodium orthovanadate leads to recovery of adduct export. Representative widefield fluorescence and corresponding DIC (inset) images of HeLa cells with 100 nM AcroB after (D) no treatment or (E) treatment with 8 mM sodium orthovanadate for 60 minutes. Imaging media was not removed (No Wash) or removed after the 16-minute timepoint and replaced with LCIS alone (Wash) or replenished with 100 nM AcroB in LCIS (Wash + replenish AcroB). Images acquired (20X magnification) over 60 minutes following initial AcroB application, LUT range 0-6000, scale bar is 64 µm. λ_{ex} = 488 nm (0.1 mW). (F) Average cellular fluorescence (CTCF) and fluorescence background curves representing n = 8 FOV (Control, No Wash), n = 6 FOV (Control, Wash), n = 6 FOV (Control, Wash + replenish AcroB), n = 7 FOV (Sodium orthovanadate, No Wash), n = 7 FOV (Sodium orthovanadate, Wash), and n = 6 FOV (Sodium orthovanadate, Wash + replenish AcroB) obtained from 20X magnification images. The legends included in the CTCF "Control" and "Sodium orthovanadate" panels also apply to the "Background" panel. Increase in Sodium Orthovanadate No Wash condition CTCF following the "wash" period is attributed to continued accumulation of cytosolic AcroB adducts during the washing period without the contribution of photobleaching that occurs during the regular imaging intervals. Values presented as mean ± SEM. Vertical bars indicate time of media washing. (G-I) Identification of the cell exported fluorescent AcroB-glutathione adduct. (G) Purification of the fluorescent adduct exported from HT-1080 cells into LCIS media following 1-hour AcroB treatment (1 µM). Single fluorescent peak identified by emission at 520 nm following excitation at 485 nm. Collected fraction indicated. (H) LC-HRMS (ESI+) identification of exported AcroB adduct purified by HPLC (G). Exact mass of 612.2486 m/z corresponds to the AcroB-glutathione adduct with additional aldehyde reduction within 5 ppm (left structure), suggesting AcroB may undergo secondary metabolism in the cell in addition to GSH conjugation. Exact mass of 592.2431 corresponds to the AcroB-glutathione adduct with aldehyde reduction and fluorine loss (characteristic of BODIPY MS) within 5 ppm (right structure). (I) Absorbance and fluorescence spectra of the cell-derived exported AcroB-glutathione adduct purified by HPLC (G).

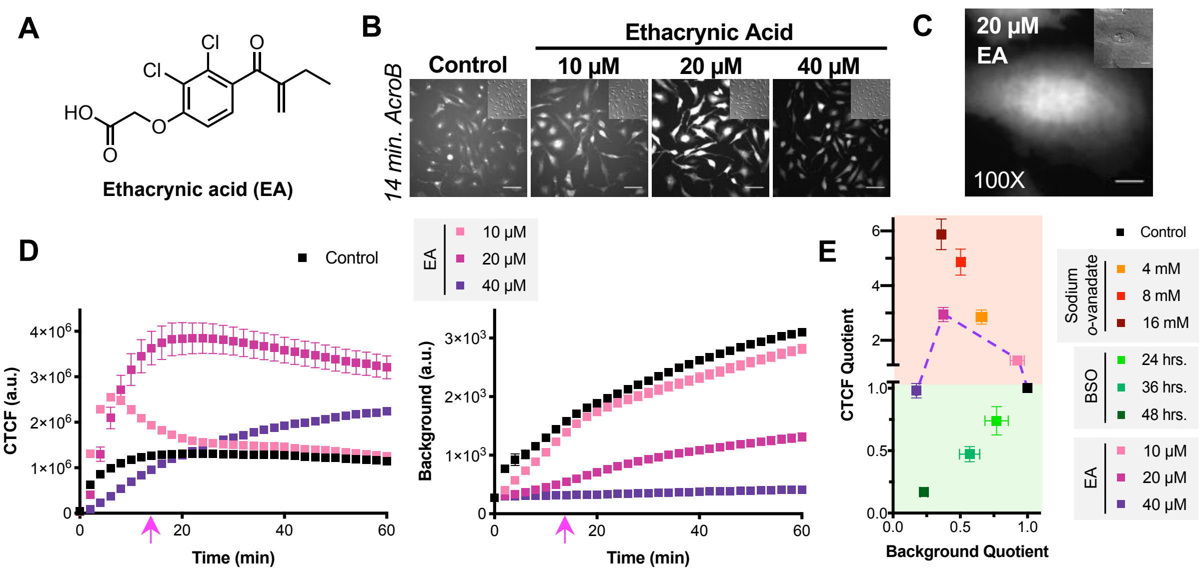

Figure S2. AcroB distinguishes between different modes of electrophile detoxification impairment following ethacrynic acid treatment. (A) Structure of ethacrynic acid. (B) Representative widefield fluorescence and corresponding DIC (inset) images of HeLa cells with 100 nM AcroB after incubation with 0-40 μM EA. Images acquired (20X magnification) over 60 minutes following AcroB application, LUT range 0-6000, scale bar is 64 μm. (C) 100X magnification widefield fluorescence and corresponding DIC (inset) images of HeLa cells with 100 nM AcroB after 2-hour treatment with 20 μM EA – image shows retention of cytosolic AcroB adducts previously observed following sodium orthovanadate treatment, Figure 2F. Image acquired 30 minutes following AcroB addition, LUT range 0-12000, scale bar is 12 μm. (D) Average cellular fluorescence (CTCF) and fluorescence background curves representing n = 11 FOV (Control), n = 11 FOV (10 μM EA), n = 6 FOV (20 μM EA) and n = 4 FOV (40 μM EA) obtained from 20X magnification images. Arrow indicates 14-min. timepoint depicted in (B). Values presented as mean ± SEM. λ_{ex} = 488 nm (0.1 mW for 20X, 0.05 mW for 100X). (E) ElectrophileQ plot including EA values obtained 20 minutes after AcroB application using data presented in (D). Line connecting Control (1.0-1.0) and EA points is a visual aid. Data point for 10 μM EA is in line with sodium orthovanadate treatment (export inhibition alone), while data points for 20 μM EA and 40 μM EA shift downwards on the ElectrophileQ plot relative to export inhibition alone, consistent with an increasing contribution of conjugation impairment with increasing [EA].

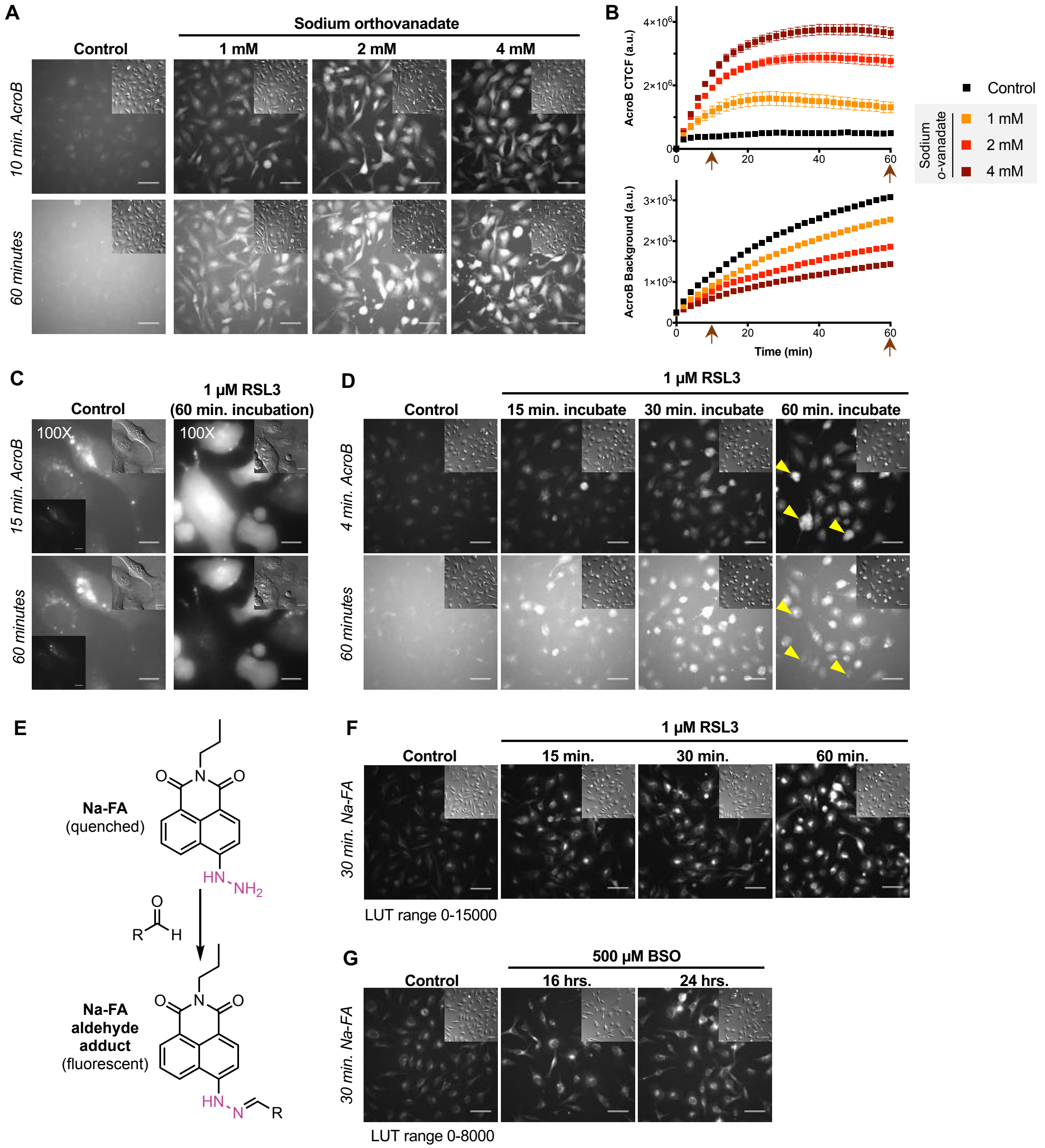

Figure S3. Supplemental images and data related to Figure 3: Ferroptosis is characterized by impaired electrophile detoxification and associated increased electrophile levels. (A-B) Characterization of AcroB sensitivity to export inhibition in HT-1080 cells – Related to Figure 3H. (A) Representative 20X widefield 100 nM AcroB fluorescence images and corresponding DIC image (inset) of HT-1080 cells following no treatment or treatment with 1-4 mM sodium orthovanadate (30 min. incubation). Images acquired over 60 minutes following AcroB addition, LUT range 0-4000, scale bar is 64 µm. λ_{ex} = 488 nm (0.1 mW). (B) Average AcroB CTCF and fluorescence background curves for sodium orthovanadate treatment representing n = 11 FOV (Control), n = 8 FOV (1 mM), n = 16 FOV (2 mM, 4 mM) obtained from 20X magnification images. Arrows indicate times depicted in (A). All values presented as mean ± SEM. (C) Example 100X AcroB fluorescence and DIC (inset) images of HT-1080 cells in control conditions or following 60-minute treatment with 1 µM RSL3 – Supplemental to Figure 3C. Scale bar is 12 µm. Control main fluorescence images LUT = 3000, inset = 10 000. RSL3 fluorescence images LUT = 0-10 000. (D) Representative 20X widefield images from 0-60-minute treatment with 1 µM RSL3 – Supplemental to Figure 3A. Images acquired over 60 minutes following

AcroB addition, LUT range 0-4000, scale bar is 64 µm. Yellow arrows highlight cells that underwent membrane rupture and AcroB fluorescence loss. (E-G) Na-FA imaging allows for relative quantification of aldehyde levels after ferroptosis induction – Related to Figure 3D,I. (E) Structure of Na-FA and structure of fluorescent Na-FA aldehyde adduct. (F) Representative 20X widefield fluorescence and corresponding DIC (inset) images with 5 µM Na-FA following 0–60-minute treatment with 1 µM RSL3. Na-FA added to cells for 30-minute incubation before shown image. Quantification presented in Figure 3D. (G) Representative 20x widefield fluorescence images with 5 µM Na-FA following 0–24-hour treatment with 500 µM BSO. Na-FA added to cells for 30-minute incubation before shown image. Quantification presented in Figure 3I. λ_{ex} = 488 nm (0.1 mW), LUT range as indicated, scale bar is 64 µm.

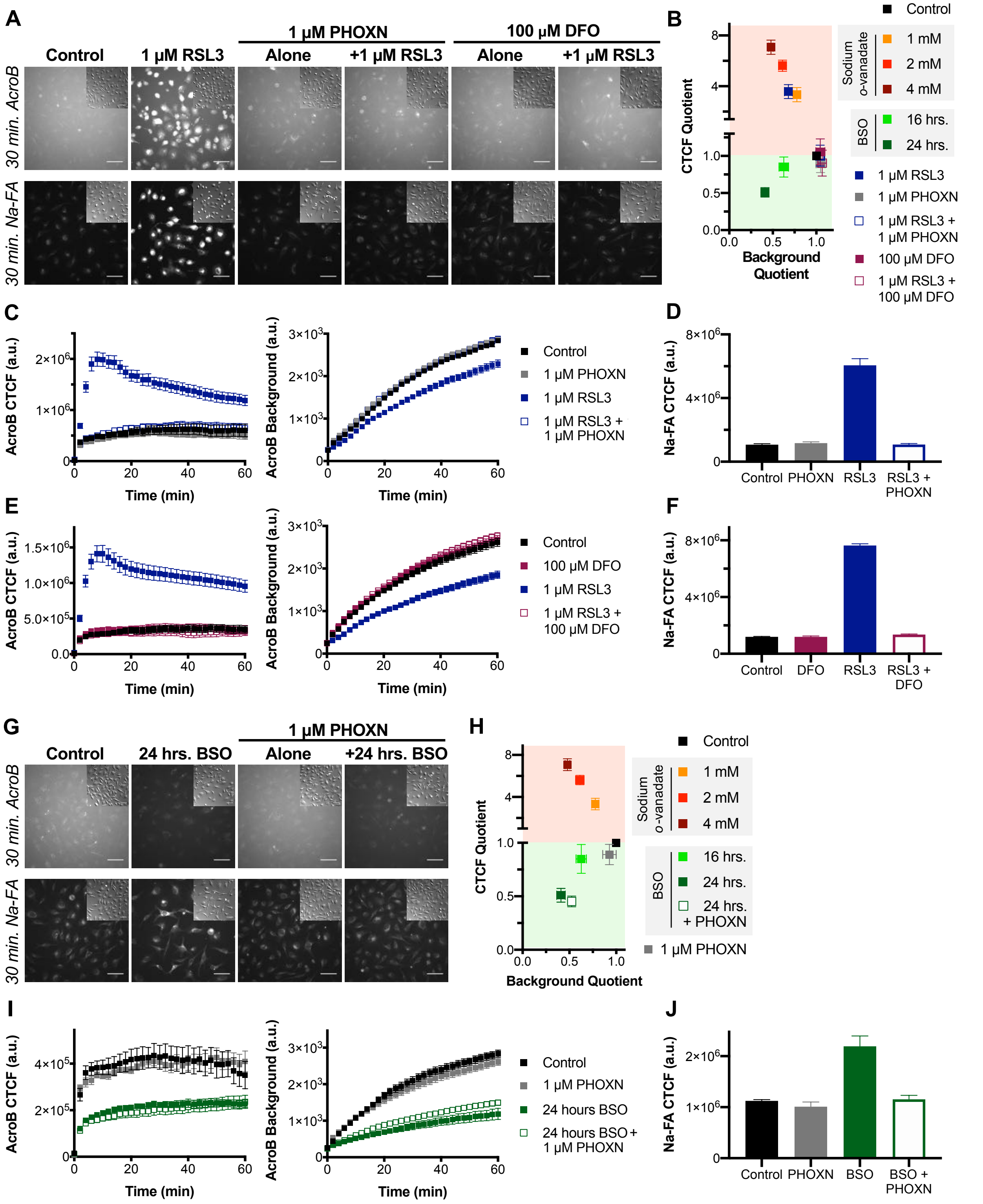

Figure S4. Treatment with ferroptosis inhibitors alters LDE metabolism following ferroptosis induction. (A-F) Inhibition of RSL3-induced ferroptosis with phenoxazine or DFO recovers electrophile detoxification capacity and level of cellular aldehydes. (A) Representative 20X widefield fluorescence images and corresponding DIC image (inset) of HT-1080 cells following no treatment, treatment with 1 μM RSL3 (60 min. incubation) and/or 1 μM PHOXN or 100 μM DFO imaged with 100 nM AcroB

(LUT range 0-4000) or 5 μM Na-FA (LUT range 0-15000). Scale bar is 64 μm. λ_{ex} = 488 nm (0.1 mW). (B) ElectrophileQ plot for HT-1080 cells obtained 20 minutes after AcroB application using data presented in (C) and (E). (C) Average AcroB CTCF and fluorescence background curves representing n = 13 FOV (Control, 1 μM PHOXN) and n = 16 FOV (1 μM RSL3, RSL3 + PHOXN) obtained from 20X magnification images. (D) Na-FA CTCF levels (5 μM, 30-minute incubation) in HT-1080 cells after indicated treatments. Average of n = 4 FOV for all conditions. (E) Average AcroB CTCF and fluorescence background curves representing n = 15 FOV (Control), n = 14 FOV (100 μM DFO), n = 16 FOV (1 μM RSL3) and n = 13 FOV (RSL3 + DFO) obtained from 20X magnification images. (F) Na-FA CTCF levels (5 μM, 30-minute incubation) in HT-1080 cells after indicated treatments. Average of n = 8 FOV for all conditions. (G-J) Inhibition of BSO-induced ferroptosis with phenoxazine recovers level of cellular aldehydes but not electrophile detoxification capacity. (G) Representative 20X widefield fluorescence images and corresponding DIC image (inset) of HT-1080 cells with/without treatment with 1 μM PHOXN and/or 500 μM BSO (24-hour incubation) imaged with 100 nM AcroB (LUT range 0-4000) or 5 μM Na-FA (LUT range 0-8000). Scale bar is 64 μm. λ_{ex} = 488 nm (0.1 mW). (H) ElectrophileQ plot for HT-1080 cells obtained 20 minutes after AcroB application using data presented in (I). (I) Average AcroB CTCF and fluorescence background curves representing n = 12 FOV (Control), n = 7 FOV (1 μM PHOXN), n = 11 FOV (500 μM BSO) and n = 16 FOV (BSO + PHOXN) obtained from 20X magnification images. (J) Na-FA CTCF levels (5 μM, 30-minute incubation) in HT-1080 cells after indicated treatments. Average of n = 8 FOV (Control), n = 6 FOV (1 μM PHOXN), n = 16 FOV (500 μM BSO) and n = 12 FOV (BSO + PHOXN). All values presented as mean ± SEM.

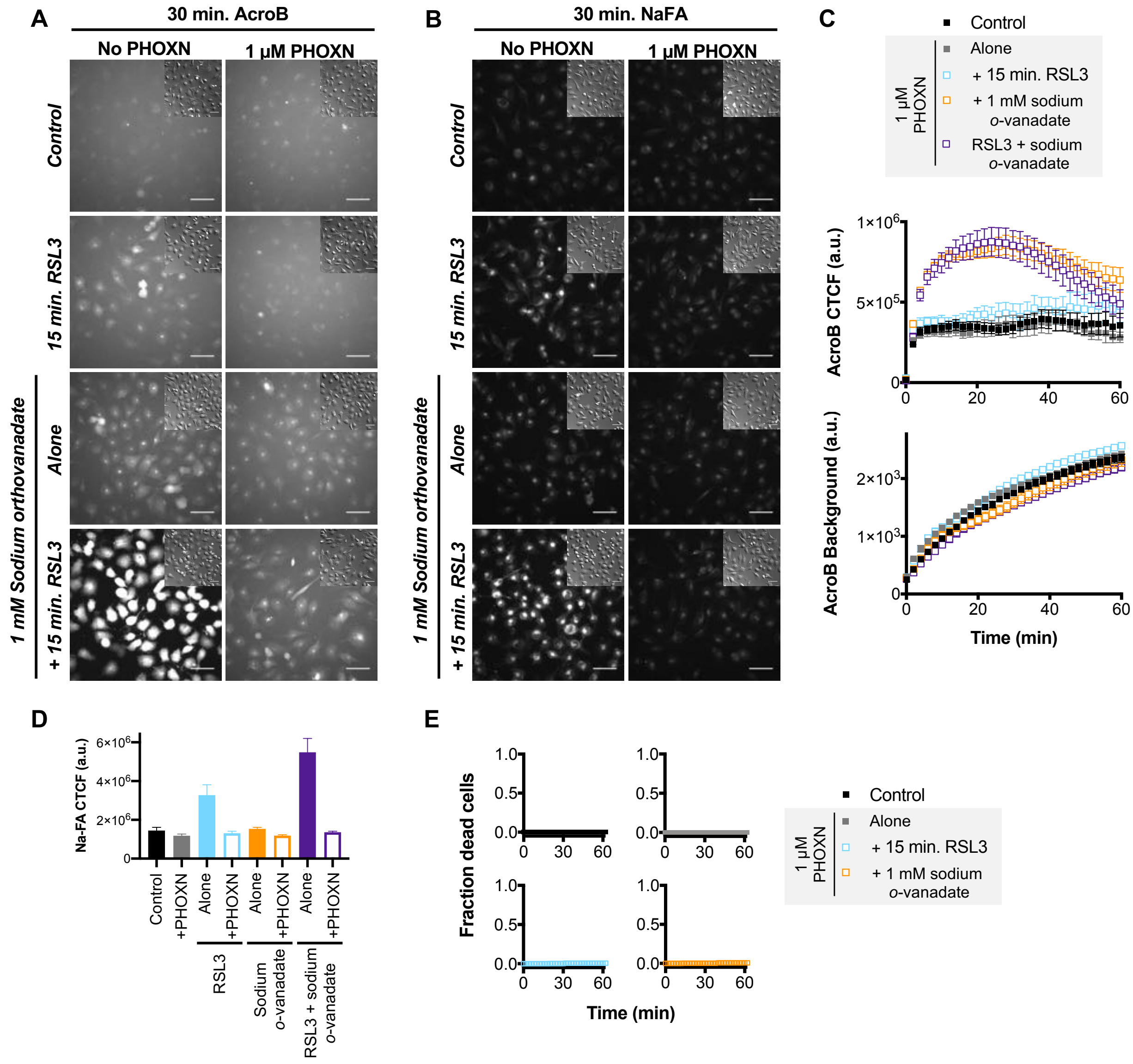

Figure S5. Sodium orthovanadate pre-treatment leads to exacerbated electrophile export impairment, aldehyde accumulation and cell death following sub-lethal RSL3 treatment that is prevented with phenoxazine. (A-B) Representative 20X widefield fluorescence images and corresponding DIC image (inset) of HT-1080 cells with/without treatment with 1 µM PHOXN, 1 µM RSL3 (15 min. incubation), and/or sodium orthovanadate (1 mM, 30 min. incubation) imaged with (A) 100 nM AcroB (LUT range 0-4000) or (B) 5 µM Na-FA (LUT range 0-15000). Scale bar is 64 µm. λ_{ex} = 488 nm (0.1 mW). (C) Average AcroB CTCF and fluorescence background curves representing n = 11 FOV (Control), n = 6 FOV (1 µM PHOXN), n = 6 FOV (RSL3 + PHOXN), n = 10 FOV (sodium orthovanadate + PHOXN) and n = 10 FOV (sodium orthovanadate + RSL3 + PHOXN) obtained from 20X magnification images. Supplemental to Figure 3C. Values presented as mean ± SEM. (D) Na-FA CTCF levels (5 µM, 30-minute incubation) in HT-1080 cells after indicated treatments. Average of n = 5 FOV (Control, 15 min. RSL3, 1 mM sodium orthovanadate, RSL3 + sodium orthovanadate) and n = 4 FOV (PHOXN conditions) obtained from 20X magnification images. (E) Fraction of dead cells as determined by 20X widefield propidium iodide (10 µM) fluorescence imaging for additional control conditions not presented in Figure 4B. Values presented as mean ± SEM.

Supplemental Movies

Movie M1. 100× widefield fluorescence of a HeLa cell in the presence of 100 nM AcroB in control conditions over 30 minutes. Scale bar represents 12 µm. LUT range is 0-5000.

Movie M2. 20× widefield fluorescence of field of view of HeLa cells in control conditions over 60 minutes. Scale bar represents 64 µm. LUT range is 0-6000.

Movie M3. 100× widefield fluorescence of HeLa cell treated for 48 hours with 500 µM BSO prior to imaging in the presence of 100 nM AcroB. Scale bar represents 12 µm. LUT range is 0-2000.

Movie M4. 100× widefield fluorescence of a HeLa cell treated for 2 hours with 8 mM sodium orthovanadate prior to imaging in the presence of 100 nM AcroB. Scale bar represents 12 µm. LUT range is 0-5000.

Movie M5. 100× widefield fluorescence of HT-1080 cells treated for 60 minutes with 1 µM RSL3 prior to imaging in the presence of 100 nM AcroB. Scale bar represents 12 µm. LUT range is 0-10000.

References

1. R. Lincoln, L. E. Greene, W. Zhang, S. Louisia and G. Cosa, *J. Am. Chem. Soc.*, 2017, **139**, 16273-16281.
2. Y. Tang, X. Kong, A. Xu, B. Dong and W. Lin, *Angew. Chem. Int. Ed.*, 2016, **55**, 3356-3359.
3. *Cold Spring Harb. Protoc.*, 2017, **2017**, pdb.rec092940.
4. J. Schindelin, I. Arganda-Carreras, E. Frise, V. Kaynig, M. Longair, T. Pietzsch, S. Preibisch, C. Rueden, S. Saalfeld, B. Schmid, J.-Y. Tinevez, D. J. White, V. Hartenstein, K. Eliceiri, P. Tomancak and A. Cardona, *Nat. Methods*, 2012, **9**, 676-682.
5. O. Gavet and J. Pines, *Dev. Cell*, 2010, **18**, 533-543.
